# Supplementary material for: Multi‐dataset identification of innovative feature genes and molecular mechanisms in keratoconus
Source: J Cell Mol Med. 2024 Sep 19;28(18):e70079. doi: 10.1111/jcmm.70079 (PMC11412914; doi:10.1111/jcmm.70079)
Supplement: Supplementary file 2 — Material S2. [file JCMM-28-e70079-s002.zip › 7.GSEA analysis/3.DBNDD1/KEGG_GLYCEROLIPID_METABOLISM.html]

Details for gene set KEGG\_GLYCEROLIPID\_METABOLISM[GSEA]

|  || Dataset | DBNDD1.group.cls#h\_versus\_l |
| Phenotype | group.cls#h\_versus\_l |
| Upregulated in class | h |
| GeneSet | KEGG\_GLYCEROLIPID\_METABOLISM |
| Enrichment Score (ES) | 0.21065839 |
| Normalized Enrichment Score (NES) | 0.7350172 |
| Nominal p-value | 0.8869396 |
| FDR q-value | 1.0 |
| FWER p-Value | 1.0 |
Table: GSEA Results Summary

  

Fig 1: Enrichment plot: KEGG\_GLYCEROLIPID\_METABOLISM      
 Profile of the Running ES Score & Positions of GeneSet Members on the Rank Ordered List

  

| SYMBOL | TITLE | RANK IN GENE LIST | RANK METRIC SCORE | RUNNING ES | CORE ENRICHMENT || 1 | GPAM | na | 1375 | 0.347 | 0.0242 | Yes |
| 2 | DGKG | na | 1713 | 0.319 | 0.0733 | Yes |
| 3 | GK2 | na | 2001 | 0.299 | 0.1201 | Yes |
| 4 | CEL | na | 2068 | 0.294 | 0.1724 | Yes |
| 5 | DGKB | na | 2719 | 0.263 | 0.2020 | Yes |
| 6 | PNLIPRP2 | na | 4332 | 0.202 | 0.1928 | Yes |
| 7 | GLYCTK | na | 5037 | 0.179 | 0.2055 | Yes |
| 8 | LIPF | na | 6674 | 0.132 | 0.1826 | Yes |
| 9 | AKR1A1 | na | 7373 | 0.116 | 0.1839 | Yes |
| 10 | DGKZ | na | 7408 | 0.115 | 0.2042 | Yes |
| 11 | DGKE | na | 8317 | 0.098 | 0.1960 | Yes |
| 12 | PNPLA3 | na | 9545 | 0.078 | 0.1749 | Yes |
| 13 | AGPAT3 | na | 9643 | 0.076 | 0.1862 | Yes |
| 14 | PNLIPRP1 | na | 9888 | 0.073 | 0.1925 | Yes |
| 15 | MBOAT1 | na | 10584 | 0.063 | 0.1842 | Yes |
| 16 | DGAT2 | na | 10922 | 0.059 | 0.1853 | Yes |
| 17 | LCLAT1 | na | 11078 | 0.057 | 0.1914 | Yes |
| 18 | AGPAT2 | na | 11102 | 0.057 | 0.2012 | Yes |
| 19 | DGKD | na | 11212 | 0.056 | 0.2084 | Yes |
| 20 | AGK | na | 11468 | 0.052 | 0.2107 | Yes |
| 21 | DGKQ | na | 11819 | 0.049 | 0.2096 | No |
| 22 | ALDH3A2 | na | 12835 | 0.040 | 0.1876 | No |
| 23 | LPL | na | 15320 | 0.020 | 0.1197 | No |
| 24 | AGPAT1 | na | 17933 | 0.003 | 0.0450 | No |
| 25 | AGPAT4 | na | 18200 | 0.001 | 0.0376 | No |
| 26 | ALDH7A1 | na | 18637 | -0.002 | 0.0254 | No |
| 27 | DGAT1 | na | 18959 | -0.004 | 0.0169 | No |
| 28 | PNLIP | na | 22147 | -0.033 | -0.0689 | No |
| 29 | ALDH1B1 | na | 22410 | -0.036 | -0.0697 | No |
| 30 | DGKH | na | 22724 | -0.040 | -0.0714 | No |
| 31 | ALDH9A1 | na | 22786 | -0.041 | -0.0657 | No |
| 32 | MBOAT2 | na | 25429 | -0.077 | -0.1276 | No |
| 33 | DGKA | na | 26087 | -0.089 | -0.1302 | No |
| 34 | GLA | na | 26169 | -0.091 | -0.1157 | No |
| 35 | MGLL | na | 26232 | -0.092 | -0.1005 | No |
| 36 | LIPG | na | 27675 | -0.123 | -0.1195 | No |
| 37 | AKR1B1 | na | 28153 | -0.134 | -0.1085 | No |
| 38 | GK | na | 28724 | -0.149 | -0.0974 | No |
| 39 | GPAT2 | na | 29726 | -0.180 | -0.0932 | No |
| 40 | DGKI | na | 31612 | -0.245 | -0.1024 | No |
| 41 | LIPC | na | 31818 | -0.255 | -0.0614 | No |
| 42 | ALDH2 | na | 33235 | -0.347 | -0.0383 | No |
| 43 | AWAT2 | na | 34088 | -0.442 | 0.0185 | No |
Table: GSEA details [plain text format]

  

Fig 2: KEGG\_GLYCEROLIPID\_METABOLISM      
 Blue-Pink O' Gram in the Space of the Analyzed GeneSet

  

Fig 3: KEGG\_GLYCEROLIPID\_METABOLISM: Random ES distribution      
 Gene set null distribution of ES for **KEGG\_GLYCEROLIPID\_METABOLISM**
